# Aging during COVID-19 in Germany: a longitudinal analysis of psychosocial adaptation

**DOI:** 10.1007/s10433-021-00655-1

**Published:** 2021-10-01

**Authors:** Anna Schlomann, Mareike Bünning, Lena Hipp, Hans-Werner Wahl

**Affiliations:** 1 Network Aging Research, Heidelberg University, Bergheimer Straße 20, 69115 Heidelberg, Germany; 2 Institute for Educational Sciences, Heidelberg University of Education, Heidelberg, Germany; 3 WZB Berlin Social Science Center, Berlin, Germany; 4 German Center of Gerontology (DZA), Berlin, Germany; 5 University of Potsdam, Potsdam, Germany; 6 Institute of Psychology, Heidelberg University, Heidelberg, Germany

**Keywords:** Coronavirus pandemic, Older adults, Satisfaction with life, Satisfaction with social relations, Age stereotypes, Longitudinal survey study

## Abstract

Existing theories of aging suggest that there may be similarities and differences in how COVID-19 impacts older people’s psychosocial adaptation compared to younger age groups, particularly middle-aged individuals. To assess the degree to which these impacts vary, we analyzed data from 3098 participants between the ages of 40 and 79 from an online survey in Germany. Data were collected at three measurement occasions between the start of the nationwide lockdown in mid-March 2020 and the end of the lockdown in early August 2020. The survey focused on everyday experiences during the COVID-19 crisis and collected various satisfaction ratings (e.g., general life satisfaction, satisfaction with family life, satisfaction with social contacts). At baseline, participants also provided retrospective ratings of satisfaction for the period before the COVID-19 crisis. In our analyses, we compared satisfaction ratings of middle-aged (40–64 years) and older individuals (65–79 years) and found that both middle-aged and older participants experienced the greatest decreases in satisfaction with social contacts, with more pronounced decreases seen in middle-aged participants. A similar pattern was observed for general life satisfaction, but the overall decreases were less pronounced in both groups compared to the decreases in satisfaction with social contacts. We also observed a partial recovery effect in all measures at the last measurement occasion, and this effect was more pronounced in older adults. Findings were also confirmed using age as a continuous variable and checking for linear and nonlinear effects of outcomes across the age range. Although ageism arose during the pandemic in the sense that older adults were labeled as a “risk group,” particularly at the start of the outbreak, we found consistently with other studies that middle-aged adults’ satisfaction decreased to a greater extent than that of older adults.

**Supplementary Information:**

The online version contains supplementary material available at 10.1007/s10433-021-00655-1.

## Introduction

As has been officially documented and reported across a large number of countries, older adults were the most severely affected of all age groups by the COVID-19 pandemic in terms of their health and lives (WHO 2020). However, not much is known about how the crisis has affected older adults’ life satisfaction and psychological adaptation compared to other age groups—middle-aged adults, in particular. As older adults were negatively stereotyped as a “risk group” during the crisis due to their chronological age (Ayalon et al. 2021; Ehni and Wahl 2020; Jiminez-Sotomayor et al. 2020), it may well be that older individuals were affected differently by the COVID-19 pandemic than younger age groups.

### Psychosocial challenges for older adults during the COVID-19 crisis

The SARS-CoV2 virus created an unexpectedly stressful situation for individuals around the globe, including both community-dwelling older adults as well as older adults living in long-term care settings (Sands et al. 2020). This article looks at community-dwelling older adults. In Germany, where we conducted our study, older adults were affected by the crisis in at least four ways. First, data on mortality caused by COVID-19 infections were regularly published and discussed by the press and media; such reports clearly indicated that survival after hospitalization, particularly after ventilator treatment, was significantly lower in older adults (Wolf-Ostermann and Rothgang 2020).

Second, the ongoing discussion on the availability of intensive care beds (ICBs) and public communications on the possible need for triage in case of a shortage of ICBs raised fears among older adults that, if infected, they would not receive optimal medical treatment and would be accorded a lower priority, due primarily to their chronological age (Ehni and Wahl 2020). Television reports on the situation in other European countries—in particular, Italy, France, and Spain—suggested that age-based triage may have been utilized due to ICB shortages. Images in the media of military trucks in Italy being used to transport scores of coffins may have exacerbated such fears among older adults in Germany (see also Cesari and Proietti 2020).

Third, the lockdown, which began in Germany in mid-March 2020, led to complete social isolation among older adults in nursing homes, but the same was also largely true for older people living in the community. Many valuable activities for older adults, such as visiting or being visited by children and grandchildren, shopping, eating out, going to cultural events, taking walks with peers, volunteering, or participating in educational programs, were out of the question. Constraints on social interactions and activities were among the greatest challenges facing older adults in the initial phase of the pandemic (Heid et al. 2021). The resulting separation from family and close friends put older adults at risk of loneliness, which can eventually lead to physical and mental health decline (Tyrrell and Williams 2020). At this point in time, it was also unclear how long the situation would last.

Fourth, older adults faced ageism and negative age stereotyping in that they were seen as the major “risk group” who had to be protected by the lockdown and who had forced younger age groups to stay home, minimize activity, and stop going to school—a development that had dramatic impacts on parents’ lives and childcare duties (Ayalon et al. 2021; Ehni and Wahl 2020).

The current paper addresses how middle-aged (40–64 years) and older adults (65–79 years) have psychologically adapted to the COVID-19 crisis in Germany covering a timespan with three measurement occasions from the point in time when the outbreak caused a country-wide lockdown to the end of the lockdown about five months later.

### Conceptual approach

To conceptually capture the pandemic’s psychosocial consequences, we draw on socio-emotional selectivity theory and stress and resilience theory. First, socio-emotional selectivity theory (Carstensen 2006; English and Carstensen 2016; see also Martin 2020) argues that if time is perceived as limited, which is typically the case in old age and may have been reinforced by the COVID-19 crisis, people value emotionally meaningful goals and relationships more than other goals in life. This activates mood-enhancing goals and reduces the willingness to accept temporary negative experiences for the sake of long-term benefits. Findings related to socio-emotional selectivity theory have shed light on a phenomenon referred to as the *positivity effect* of aging (Scheibe and Carstensen 2010). The positivity effect relates to a shift that occurs with advancing age toward paying attention to or remembering positive as opposed to negative information.

A second theoretical lens through which to analyze the crisis’s impact on individual well-being is stress theory (Lazarus 1993). The COVID-19 crisis represented a major physical and psychological health threat to populations worldwide, making coping and adaptational efforts necessary (Aravena et al. 2020). Recent research findings suggest that the COVID-19 pandemic caused various stress-related symptoms and anxiety for many people around the world (e.g., Casagrande et al. 2020; González-Sanguino et al. 2020). From the perspective of stress theory, it is important to note that the crisis came without warning and did not allow time to anticipate or prepare. Established stress research has shown that anticipation and prediction may increase feelings of control over stressors and thus reduce overall stress (Lazarus 1993). However, more recent work has also suggested that stress experiences in day-to-day life may increase “when you see it coming” (Neubauer et al. 2018). That said, the COVID-19 crisis may at least be partially similar to a natural disaster and may have similar consequences. This is relevant because there are findings suggesting that older adults exhibit higher resilience during natural disasters and have more positive outcomes than other age groups (Eshel et al. 2016; Rafiey et al. 2016; but see Parker et al. 2016 for opposing evidence). A recent study also found that older adults showed high levels of resilience and perceived themselves to be coping well during the initial phase of the COVID-19 pandemic (Fuller and Huseth-Zosel 2021). In addition, previous research on the so-called well-being paradox supports the notion that general life satisfaction is high in older adults and relatively robust against stress experiences (Diehl et al. 2020; Kunzmann et al. 2000).

### Hypotheses

As suggested by the conceptual models applied to the COVID-19 crisis as outlined above, we examined two hypotheses relating to satisfaction ratings and explore possible recovery effects over the course of the pandemic. First, applying socio-emotional selectivity theory and the positivity effect to the COVID-19 crisis, we expected the crisis to have caused stronger decreases in satisfaction with social contacts and family life than in satisfaction with life in general among both middle-aged and older adults. Given the positivity effect in old age, we also expected the decreases in satisfaction with social contacts to be more pronounced among middle-aged adults than among older adults. Second, we expected a decrease in general life satisfaction as a result of the stress caused by the COVID-19 crisis that was of similar magnitude among both middle-aged and older adults. We also explored whether recovery effects occurred during the lockdown that followed the pandemic’s outbreak in Germany as suggested by the psycho-adaptational literature on critical life events (Luhmann et al. 2012; Schilling and Wahl 2006).

## Research design and methods

### Sample and design

In our analyses, we drew on three waves of survey data gathered in an online survey to capture individuals’ everyday experiences during the COVID-19 lockdown in Germany. The survey started one week after Germany went into a nationwide lockdown, which entailed the closure of schools, shops, restaurants, and businesses and the prohibition of public gatherings. Participants learned about the study through e-mail lists, newspaper announcements, and instant messaging services. Participants who agreed to be surveyed again were invited to participate in a follow-up survey 3.5–4 weeks after they completed the first questionnaire and again ten weeks after the first interview. The first wave of data collection ran between March 23, 2020, and May 11, 2020. The second wave of data collection (April 20–June 14, 2020) coincided with the tentative lifting of the lockdown, including the limited reopening of schools and shops. The third wave (June 3–August 2, 2020) coincided with further steps toward reopening, including the decision to restart regular schooling after the summer holidays and the government’s presentation of its economic stimulus package.

The entire sample amounted to 14,754 individuals aged 18 years and older in survey wave 1 (W1), 7573 in wave 2 (W2), and 6397 in wave 3 (W3). In our analytical sample, we included those 3098 participants aged 40–79 years who participated in all three waves (see Hipp and Bünning 2020 for a similar approach). Data from participants aged 80 years and older were excluded from our analyses due to the relatively low sample size and the selectivity of “onliners” in this age category (see, for example, Schlomann et al. 2020). Due to our theoretical interest, our analyses distinguished between middle-aged individuals (40–64 years) and older individuals (65–79 years). More than 13% of the respondents in the analytical sample (*N* = 413) were aged between 65 and 79 years. Besides the differences in chronological age between the two groups, these two age groups differ substantially with regard to their labor force participation and their childcare and other care activities.

As can be seen in Table 1, the majority of participants were female (73%), partnered (73%), and highly educated (77% of all participants had a university degree). In total, 85% of all participants were employed (age group 40–64: 93%, age group 65–79: 26%). Self-rated pre-pandemic physical health was 2.10 on a five-point scale in the whole sample.

**Table 1:** Descriptive statistics (at t1)

	All			Age 40–64			Age 65–79		
	Mean	SD	*N*	Mean	SD	*N*	Mean	SD	*N*
Age 65–79	0.13	0.34	3098						
Women	0.73	0.45	3090	0.75	0.43	2677	0.59	0.49	413
Partnered	0.73	0.44	3070	0.74	0.44	2664	0.68	0.47	406
University degree	0.77	0.42	3058	0.77	0.42	2654	0.75	0.43	404
Working	0.85	0.36	3016	0.93	0.25	2628	0.26	0.44	388
Self-rated pre-pandemic physical health^a^	2.10	0.82	3091	2.10	0.81	2679	2.09	0.84	412
Town size > 50,000 inhabitants	0.67	0.47	3082	0.68	0.47	2671	0.61	0.49	411
*N*	3098			2685			413		

^a^Five-point scale, higher values indicate poorer health

The sociodemographic characteristics of individuals who participated in all three survey waves were very similar to those of the full W1 sample (see Table 1—Supplement). To assess respondents’ situations before the COVID-19 crisis began, we included several retrospective questions (t0). To minimize inaccurate answering behaviors due to the high cognitive load of retrospective questions (Yan and Tourangeau 2007) and social desirability biases (Jaspers et al. 2009), we ensured that questions about the respondents’ pasts were short, easy to understand, and referred to a specific anchor point (the pre-pandemic period) that was only a short time (2–6 weeks) before data collection. All of these factors have been shown to increase recall accuracy, even for subjective assessments such as ratings of health status and subjective well-being (Barsky 2002), and recall accuracy was found to be independent of respondents’ age or educational background (Hipp et al. 2020).

### Measures

#### Psychosocial adaptation

Respondents were asked about their satisfaction (measured on a seven-point Likert scale) with family life, the quality of social contacts (including contact by telephone, e-mail, and other media), and satisfaction with life in general.^1^ All three satisfaction ratings were single-item questions. Single-item measures are a commonly used and cost-effective method in survey research, and single-item measures of life satisfaction have been found to be reasonably valid (see Richter et al. 2017, for an overview).

#### Covariates

The following covariates were included in the multivariate analyses: gender (female/male), presence of partner in the household (dummy), level of education (dummy variable indicating university degree), whether the person was working (dummy), self-reported physical health prior to the pandemic (one item, five-point Likert scale, adapted from the German Socio-Economic Panel), and the size of the town or city where respondents reside (greater/fewer than 50,000 residents). The presence of a partner and employment status was assessed in each wave; the remaining variables were assessed at t1.

### Data analysis

The survey data were analyzed using OLS regressions with clustered, robust standard errors using four points of measurement (t0 [retrospective assessment] to t3). We conducted separate analyses for each life domain. To test whether the coefficients significantly differed by age group and time point, we included interaction effects for age and time point. To compare coefficients across life domains, we used seemingly unrelated regressions and χ2-tests. To assess the robustness of our results, in particular the theoretically derived age group comparison, we conducted the following robustness checks: First, we used age as a continuous variable (age and age squared) instead of distinguishing between the two age groups. Second, we replicated the analyses based on the full sample of 6510 respondents who participated in wave 1 and using full information maximum likelihood (Enders 2001).

## Results

**Fig. 1:**
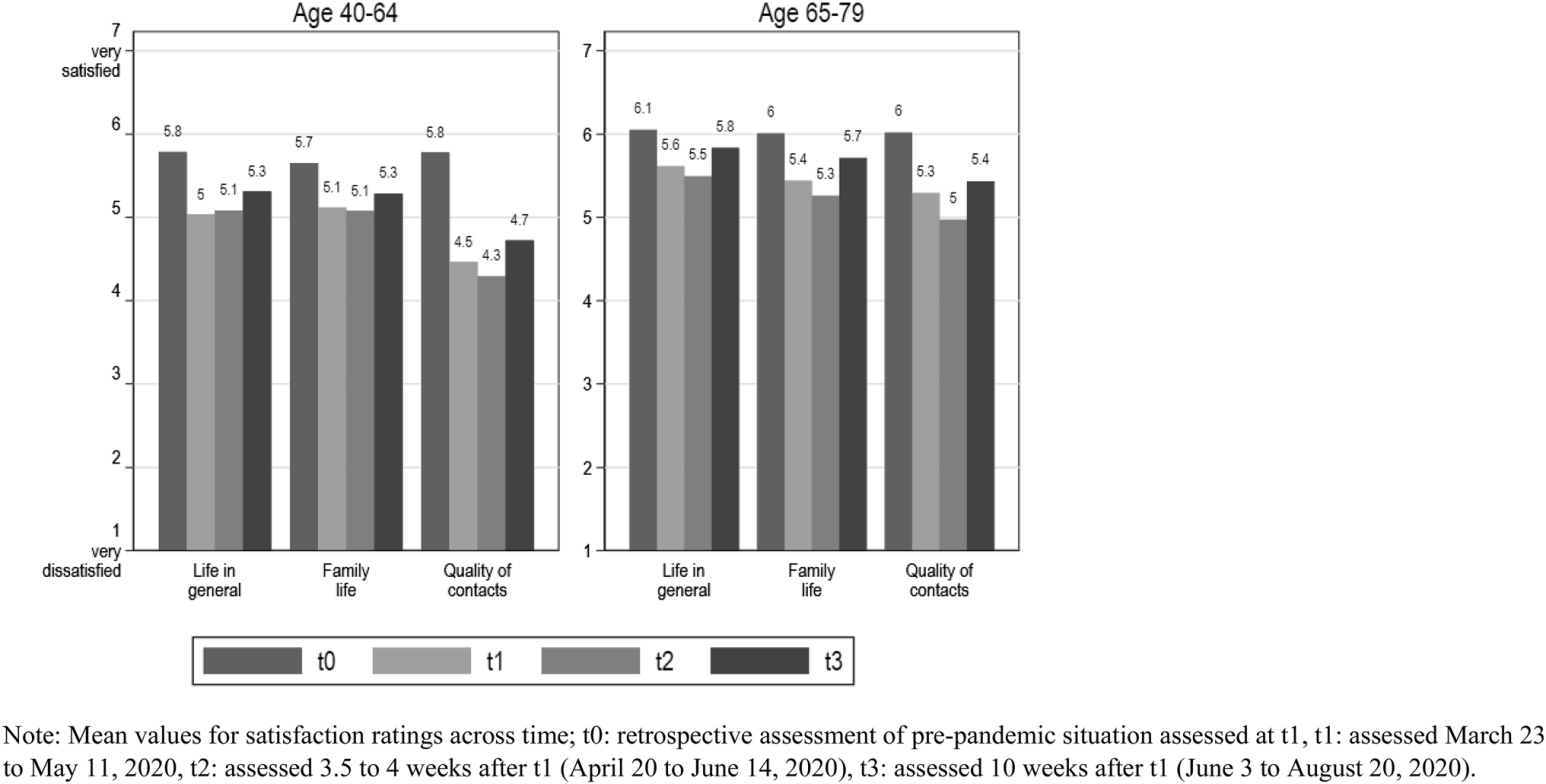
Sample means of satisfaction ratings by age group and measurement occasion

We start by presenting mean levels of all satisfaction ratings across the four time points for the two age groups separately (Fig. 1). Satisfaction ratings decreased in both age groups for general life satisfaction and for satisfaction with family life and quality of social contacts at the beginning of the pandemic and recovered to some extent in the last observation period.

### Psychosocial adaptation across time

Findings from the OLS regressions are summarized in Table 2. Respondents from both age groups reported significantly higher levels of satisfaction with life in general, family life, and the quality of social contacts before the beginning of the pandemic (t0) than during the first weeks of lockdown (reference category).

**Table 2:** OLS regressions on satisfaction with different domains of life using t1 as reference

	Life		Family		Contacts	
	40–64	65–79	40–64	65–79	40–64	65–79
t0	0.76^***^	0.43^***^	0.54^***^	0.50^***^	1.34^***^	0.73^***^
	[0.71, 0.81]	[0.33, 0.53]	[0.48, 0.60]	[0.37, 0.63]	[1.26, 1.41]	[0.59, 0.88]
t2	0.04	− 0.09	− 0.04	− 0.17	− 0.18^***^	− 0.28^**^
	[− 0.00, 0.09]	[− 0.25, 0.06]	[− 0.10, 0.02]	[− 0.36, 0.01]	[− 0.24, − 0.11]	[− 0.45, − 0.11]
t3	0.28^***^	0.21^**^	0.18^***^	0.24^**^	0.27^***^	0.17
	[0.23, 0.33]	[0.06, 0.36]	[0.12, 0.24]	[0.06, 0.42]	[0.20, 0.34]	[− 0.00, 0.34]
Women	− 0.12^**^	− 0.06	− 0.04	− 0.23^*^	0.11^*^	0.06
	[− 0.20, − 0.03]	[− 0.26, 0.13]	[− 0.14, 0.06]	[− 0.45, − 0.01]	[0.02, 0.21]	[− 0.16, 0.28]
Partnered	0.21^***^	0.05	0.53^***^	0.38^**^	− 0.27^***^	− 0.36^**^
	[0.12, 0.30]	[− 0.16, 0.25]	[0.42, 0.63]	[0.13, 0.64]	[− 0.37, − 0.18]	[− 0.60, − 0.11]
University degree	0.03	− 0.06	− 0.01	− 0.11	− 0.11^*^	− 0.09
	[− 0.06, 0.13]	[− 0.28, 0.16]	[− 0.12, 0.09]	[− 0.36, 0.14]	[− 0.21, − 0.01]	[− 0.33, 0.15]
Working	0.18^*^	− 0.15	0.06	− 0.13	0.11	0.00
	[0.01, 0.35]	[− 0.34, 0.05]	[− 0.12, 0.24]	[− 0.36, 0.11]	[− 0.06, 0.29]	[− 0.21, 0.21]
Health	− 0.39^***^	− 0.28^***^	− 0.35^***^	− 0.24^***^	− 0.18^***^	− 0.15^*^
	[− 0.44, − 0.34]	[− 0.39, − 0.16]	[− 0.41, − 0.29]	[− 0.38, − 0.11]	[− 0.24, − 0.13]	[− 0.28, − 0.03]
Town size > 50,000	− 0.11^*^	− 0.07	− 0.10^*^	0.02	− 0.09^*^	0.01
	[− 0.19, − 0.02]	[− 0.27, 0.12]	[− 0.19, − 0.00]	[− 0.21, 0.25]	[− 0.19, − 0.00]	[− 0.22, 0.23]
Constant	5.78^***^	6.43^***^	5.61^***^	5.98^***^	5.09^***^	5.86^***^
	[5.50, 6.07]	[5.90, 6.96]	[5.31, 5.91]	[5.35, 6.61]	[4.80, 5.38]	[5.23, 6.50]
*N* (persons)	2637	402	2636	400	2637	402
*N* (observations)	10,188	1509	10,044	1486	10,190	1514
*R*^2^	0.12	0.06	0.09	0.08	0.15	0.09

Coefficients stem from OLS regressions with clustered, robust standard errors; confidence intervals provided in parentheses

*p < 0.05; **p < 0.01; ***p < 0.001

Comparing the size of the coefficients between age groups, we found decreases in general life satisfaction and satisfaction with the quality of social contact to be more pronounced among *middle-aged* adults than *older* adults. In fact, coefficients were almost twice as high in the middle-aged group, and the differences in the size of the coefficients were statistically significant (see Table 2—Supplement). Satisfaction with family life, by contrast, decreased to a similar extent in both age groups.

Comparing the changes in satisfaction across different areas of life, we found that decreases in satisfaction with contact quality were most pronounced in both age groups. Among middle-aged respondents, decreases in general life satisfaction came second, followed by decreases in satisfaction with family life, whereas older respondents’ satisfaction with both areas decreased to a similar extent (see Table 4—Supplement).

In addition, there was little evidence of recovery at t2 (in the middle of the lockdown period) compared to t1. In contrast, older respondents’ satisfaction with all three life domains tended to deteriorate further, as did middle-aged respondents’ satisfaction with social contacts. Middle-aged respondents’ satisfaction with life in general and family life remained stable. Yet we observed partial recovery toward the end of the lockdown (hence, approximately four months after the start of the lockdown). Although both older and middle-aged respondents reported higher levels of life satisfaction and satisfaction with family and social contacts at t3 than at t1, older adults had a stronger recovery in general life satisfaction and satisfaction with social contacts than middle-aged adults when comparing their ratings at t3 to the pre-pandemic period (t0). Recovery in satisfaction with family life was similar for both age groups (see Table 3—Supplement). Note, however, that satisfaction ratings in all three dimensions remained considerably below pre-pandemic levels.

### Robustness checks

**Fig. 2:**
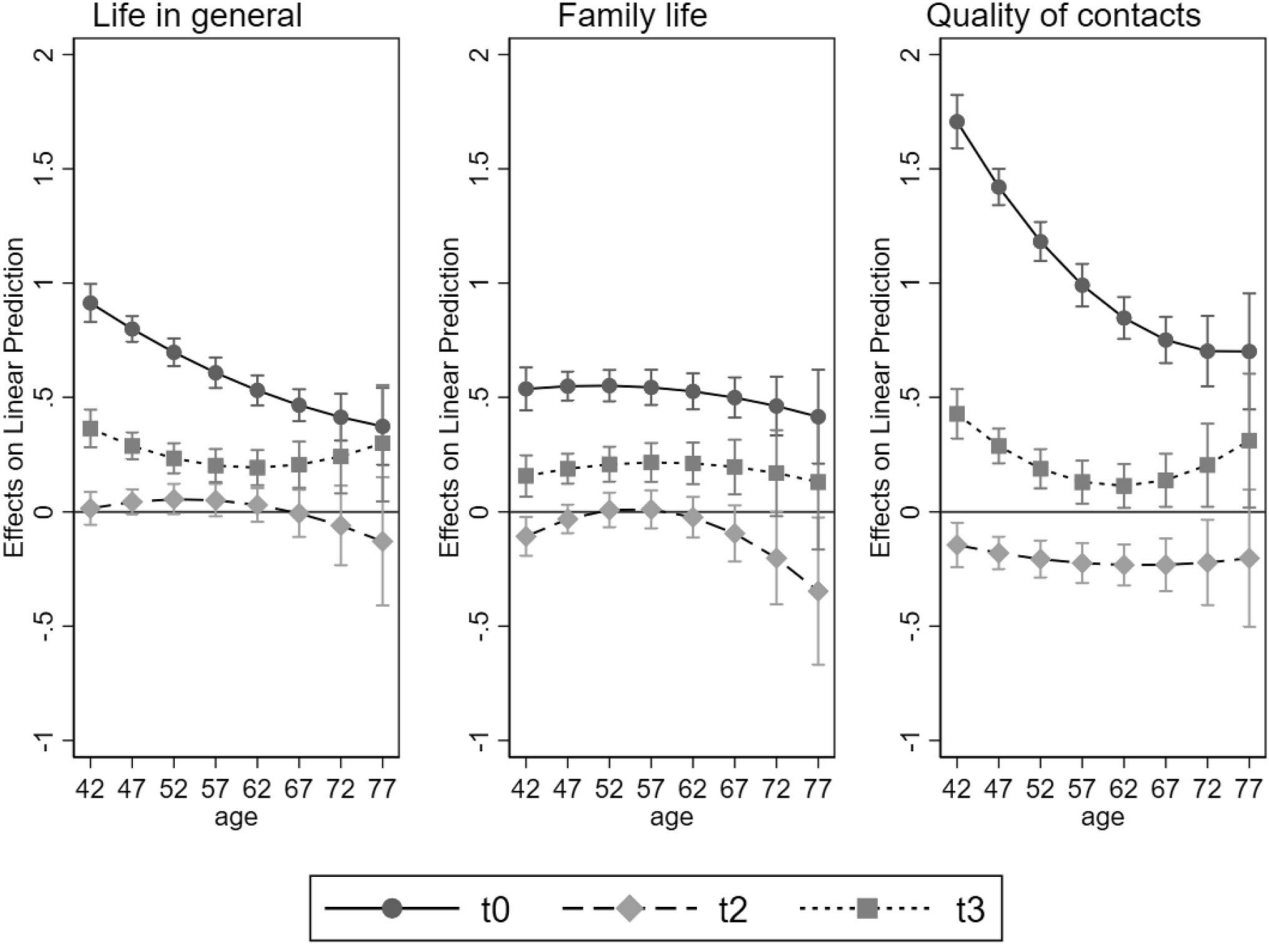
OLS regressions on satisfaction with different domains of life using age as continuous variable. *Note* Predicted changes in satisfaction ratings compared to t1 across age at three time points; t0: retrospective assessment of pre-pandemic situation assessed at t1, t1: assessed March 23–May 11, 2020, t2: assessed 3.5–4 weeks after t1 (April 20–June 14, 2020), t3: assessed 10 weeks after t1 (June 3–August 20, 2020). Age is measured by a linear and squared term. The models additionally include gender, partnership status, employment status, health, and size of town where respondents reside

To assess the robustness of our findings, we replicated the analysis using a continuous measure of age (linear and squared). Figure 2 displays changes in all three life domains across the three waves over time (taking t1 as the reference category). The younger the respondents were, the greater their decrease in life satisfaction at the onset of the lockdown (t0 vs. t1, almost linear relationship). Between t1 and t2, life satisfaction remained unchanged across the entire life span (95% confidence intervals always include 0). Increases in life satisfaction at t3 (compared to t1) were somewhat more pronounced at both ends of the age distribution than at the middle of the age distribution. While the oldest individuals returned to pre-pandemic levels of life satisfaction at t3, life satisfaction of respondents aged 67 years and younger remained considerably below pre-pandemic levels.

Decreases in satisfaction in family life between t0 and t1 were similar across the entire age span and continued to decrease at t2. Decreases at t2 were most pronounced for the oldest respondents (but note the large confidence intervals). At t3, satisfaction with family life increased again to similar degrees across the entire life span, but remained below the pre-pandemic level (t0).

For satisfaction with the quality of social contacts, the younger the respondents, the more pronounced the decrease in satisfaction between t0 and t1. The association was not linear, however, and leveled off in higher age groups, i.e., there were no differences between 60- and 79-year-olds. At t2, satisfaction with the quality of social contacts decreased further and did so to a similar degree across the entire age span. At t3, satisfaction with the quality of social contacts increased again across the entire age distribution. These increases were nonlinear and more pronounced at both ends than in the middle of the age distribution. For all ages, however, satisfaction levels at t3 remained below pre-pandemic levels, with a particularly large gap among the youngest respondents.

In addition, we replicated our original analyses using the full sample of respondents who participated in the survey at least once and full information maximum likelihood estimation (FIML, see Table 5—Supplement). The results of these analyses are similar to those presented in Table 2, with the exception of life satisfaction among the younger age group, which increased somewhat at t2 compared to t1 in the FIML (but not in the OLS) models.

## Discussion

This study explored differences in satisfaction with social life and life in general between middle-aged individuals (40–64 years) and older individuals (65–79 years) during the COVID-19 pandemic in Germany. Based on an online survey with more than 3000 respondents, we found that psychological adaptation to the COVID-19 pandemic differed to some extent between middle-aged and older individuals and that the reactions to the lockdown were contingent on the satisfaction domain as well as age.

In line with our first hypothesis, we observed the most pronounced decreases in satisfaction with the quality of social contacts and found that the decrease was more pronounced among middle-aged than older adults. In addition, the recovery in satisfaction ratings toward the end of the lockdown was stronger among older adults. More precisely, we observed a larger recovery in older adults’ satisfaction with social contacts compared to middle-aged individuals, with a particularly low level of satisfaction among the youngest persons in our sample. Still, the satisfaction levels remained below the pre-pandemic levels across the age range.

These findings suggest that the positivity effect as described by socio-emotional selectivity theory (Carstensen 2006; English and Carstensen 2016) may have protected older adults from experiencing or suffering from social isolation to some extent. Older adults tend to process negative information (such as the pandemic) less intensively than younger individuals due to their reduced future time perspective and may have a stronger tendency to emphasize positive side effects of the COVID-19 pandemic and to generally take a more positive view. In addition, older adults’ higher psychosocial resilience to challenges arising from COVID-19 may result from their accumulated life experiences (Staudinger 1999) and higher flexibility in downsizing social aspirations (Brandtstädter 2006); both processes may lead to higher satisfaction even in disadvantageous social situations. This may also indicate higher psychosocial resilience of older adults during the COVID-19 pandemic, as has been shown previously for natural disasters (Eshel et al. 2016; Rafiey et al. 2016) and which is consistent with recent findings on the COVID-19 pandemic. In a US-based study, Carney et al. (2021) reported a significant interaction effect between age and perceived COVID-19 disruption on stress and negative affect, indicating a less negative impact on well-being in increased age (see also Fuller and Huseth-Zosel 2021). In their analyses of German Socio-Economic Panel data, Entringer and Kröger (2020) found that loneliness increased among both younger and older age groups but that the increase was less pronounced among older adults. Similar trends in the results were found in studies by Luchetti et al. (2020) in the USA and Bu et al. (2020a; 2020b) in the UK, but the comparison group in this work was younger (18–29 years) than in our study (40–64 years).

In accordance with our second hypothesis, we also found decreases in general life satisfaction among both middle-aged (40–64 years) and older participants (65–79 years) compared to before the crisis (t1–t0). Again, the decrease was more pronounced among middle-aged adults. Only the oldest participants in our analytical sample returned to pre-pandemic levels of satisfaction with life in general. Besides the aforementioned explanations referring to positivity and resilience-like factors (Eshel et al. 2016; Rafiey et al. 2016; Fuller and Huseth-Zosel 2021), the established well-being paradox in old age, also labeled the “stability despite loss” paradigm (Kunzmann et al. 2000), may help in explaining the age-differential impact of the COVID-19 crisis on general life satisfaction.

An important feature of our research design was the inclusion of a second and third measurement point during the lockdown period in Germany. Interestingly, we observed no recovery in any satisfaction domain between t1 (the start of the first lockdown in Germany) and t2 (the phase during which lockdown measures began to be lifted). Instead, satisfaction with the quality of social contacts deteriorated even further across both age groups in the early months of the lockdown. We observed some recovery for both age groups and across life domains only at t3, hence about 4–5 months after the baseline, when life in Germany had returned to close to “normal” but satisfaction ratings remained largely below pre-pandemic levels.

While our survey provided us with a large number of participants throughout Germany and allowed us to track developments over time starting immediately after the first lockdown measures went into effect in March 2020, there are also a few potential biases of online surveys with voluntary participation that need to be discussed. First, although we included a broad range of covariates in our analyses to address the fact that our data came from a nonprobability online survey, and the fact that different sociodemographic groups varied in their likelihood to participate, our estimates may nonetheless be biased with regard to education, gender, and in particular, the usage of the technology necessary for an online study.

Second, intervening variables and unobserved characteristics such as attitudes and compensation strategies could not be considered as covariates because they were not included in the survey study. However, other empirical findings suggest that individuals were affected in different ways by psychological distress and anxiety caused by the COVID-19 pandemic. For example, spiritual well-being was a protective factor for depression and anxiety caused by the COVID-19 pandemic, whereas higher loneliness was associated with a more negative psychological impact (González-Sanguino et al. 2020). These possible effects should be considered in more detail in future studies applying longitudinal approaches.

Third, our analyses only included relatively healthy community-dwelling older adults, who experienced much less dramatic social consequences during the lockdown than older people in long-term care facilities. Thus, we have not addressed the very high-risk constellation of factors that affected people living in nursing homes (European Centre for Disease Prevention and Control 2020), and we cannot make any statements about older people who receive a high level of care in their private homes. Moreover, we did not include individuals aged 80 years and older in order to avoid further selection biases. It would, however, be very important to also consider adults in nursing homes and people of very old age in future COVID-19 studies on psychosocial adaptation due their very different physical and mental abilities and thus their reduced resource status (Baltes and Smith 2003).

Fourth, our findings with regard to the respondents’ retrospective assessments of their pre-pandemic well-being (t0) should also be interpreted cautiously. Despite the fact that asking retrospective questions has been increasingly used to provide a more comprehensive view of earlier phases of the life course in surveys on middle-aged and older adults (Börsch-Supan and Schröder 2011), debate continues on the measurement quality of retrospective assessment due to its proneness to inaccuracies and answering biases (Jürges 2007). In the case of this study, however, our results are most likely to be conservative, as research on retrospective questions during COVID-19 found that respondents tend to remember their past as more similar to their present (and not more idealized) when the experience or feeling of interest has changed between the time of the interview and the time point of interest (Hipp et al. 2020).

In conclusion, although ageism in the form of labeling older adults as “the risk group” occurred during the pandemic and might have caused psychological harm (Ayalon et al. 2021; Ehni and Wahl 2020), the current study found that both middle-aged and older individuals experienced decreases in a number of satisfaction ratings. However, in accordance with other large longitudinal studies, the decreases were more pronounced in middle-aged adults than in older adults. Furthermore, older adults showed a greater recovery in the general domain and in satisfaction with quality of social contacts compared to middle-aged adults.

The policy implications that can be derived from these findings can be summarized as follows: First, older adults showed a relatively high level of psychosocial resilience and recovery during the COVID-19 pandemic, which means that they should not be patronized and labeled as “risk groups”—not even with the best of intentions. It is important not only to protect older persons but to extend concerns about their well-being to society as a whole. Public decision makers and the media should therefore take more action to consider the heterogeneity in the group of the “silver agers” and “baby boomers” and should stress intergenerational solidarity.

Second, the large decrease in satisfaction with social contacts may indicate that older as well as middle-aged individuals may run into the risk of loneliness, which may eventually negatively affect their physical and mental health (see also Tyrrell and Williams 2020). The provision of comprehensive information about measures (e.g., vaccinations) may therefore help to define a future time perspective and buffer negative effects. Still, the long-term effects have not yet been studied, and future research will be needed to address the issues of psychosocial adaptation and possible negative effects of the pandemic over the life course.

## Supplementary Information

Below is the link to the electronic supplementary material.

Supplementary file 1 (DOCX 44 KB)

## Data Availability

All data are available at https://doi.org/10.7802/2042. Replication files for the analyses are available in the Supplementary Material for reviewers.

## Footnotes

1 Additionally, we collected data on respondents’ satisfaction with work (among the employed), their relationship and their division of household labor (among partnered persons), as well as satisfaction with neighborhood solidarity. In this paper, however, our focus is on the social domain and on satisfaction with life in general.
