# Supplementary material for: Aging during COVID-19 in Germany: a longitudinal analysis of psychosocial adaptation
Source: Eur J Ageing. 2021 Oct 1;19(4):1077–86. doi: 10.1007/s10433-021-00655-1 (PMC8485773; doi:10.1007/s10433-021-00655-1)
Supplement: Supplementary file 1 — (DOCX 44 KB) [file 10433_2021_655_MOESM1_ESM.docx]

**Supplementary Data**

**Table 1—Supplement: Selectivity of Sample W1-W3 Compared to Full W1 Sample**

|  | | Participants Wave 1 | | | | Participants Wave 1 to 3 | | |
| --- | --- | --- | --- | --- | --- | --- | --- | --- |
|  | mean | | sd | N | mean | | sd | N |
| Age 65-79 | 0.11 | | 0.31 | 6510 | 0.13 | | 0.34 | 3098 |
| Women | 0.69 | | 0.46 | 6454 | 0.73 | | 0.45 | 3090 |
| Partnered | 0.74 | | 0.44 | 6420 | 0.73 | | 0.44 | 3070 |
| University degree | 0.74 | | 0.44 | 6370 | 0.77 | | 0.42 | 3058 |
| Working | 0.87 | | 0.34 | 6273 | 0.85 | | 0.36 | 3016 |
| Self-rated pre-pandemic physical health | 2.11 | | 0.82 | 5112 | 2.10 | | 0.82 | 3091 |
| Town size >50,000 inhabitants | 0.65 | | 0.48 | 6456 | 0.67 | | 0.47 | 3082 |
| *N* | 6510 | |  |  | 3098 | |  |  |

**Table 2—Supplement: OLS Regressions on Satisfaction with Different Domains of Life**

**Using t1 as Reference: Pooled Model**

|  | Life | Family | Contacts |
| --- | --- | --- | --- |
| Age 65-79 | 0.64^***^ | 0.41^***^ | 0.87^***^ |
|  | [0.46,0.82] | [0.21,0.62] | [0.68,1.07] |
| t0 | 0.76^***^ | 0.54^***^ | 1.34^***^ |
|  | [0.71,0.81] | [0.48,0.60] | [1.26,1.41] |
| t2 | 0.04 | -0.04 | -0.18^***^ |
|  | [-0.00,0.09] | [-0.10,0.02] | [-0.24,-0.11] |
| t3 | 0.28^***^ | 0.18^***^ | 0.27^***^ |
|  | [0.23,0.33] | [0.12,0.24] | [0.20,0.34] |
| t0 * Age 65-79 | -0.32^***^ | -0.04 | -0.60^***^ |
|  | [-0.43,-0.21] | [-0.18,0.10] | [-0.76,-0.44] |
| t2 * Age 65-79 | -0.13 | -0.13 | -0.10 |
|  | [-0.30,0.03] | [-0.33,0.06] | [-0.28,0.08] |
| t3 * Age 65-79 | -0.07 | 0.06 | -0.11 |
|  | [-0.23,0.09] | [-0.13,0.25] | [-0.29,0.08] |
| Women | -0.10^**^ | -0.07 | 0.11^*^ |
|  | [-0.18,-0.03] | [-0.16,0.02] | [0.02,0.20] |
| Partnered | 0.19^***^ | 0.51^***^ | -0.28^***^ |
|  | [0.10,0.27] | [0.41,0.61] | [-0.37,-0.19] |
| University degree | 0.02 | -0.03 | -0.11^*^ |
|  | [-0.07,0.10] | [-0.13,0.07] | [-0.20,-0.01] |
| Working | 0.08 | 0.00 | 0.08 |
|  | [-0.06,0.21] | [-0.14,0.14] | [-0.05,0.22] |
| Health | -0.38^***^ | -0.34^***^ | -0.18^***^ |
|  | [-0.43,-0.33] | [-0.39,-0.29] | [-0.23,-0.13] |
| Town size >50,000 | -0.10^*^ | -0.08 | -0.08 |
|  | [-0.18,-0.02] | [-0.17,0.01] | [-0.17,0.01] |
| Constant | 5.86^***^ | 5.65^***^ | 5.10^***^ |
|  | [5.61,6.11] | [5.38,5.92] | [4.84,5.36] |
| N(persons) | 3039 | 3036 | 3039 |
| N(observations) | 11697 | 11530 | 11704 |
| R2 | 0.13 | 0.10 | 0.15 |

Note: Coefficients stem from OLS regressions with clustered, robust standard errors; confidence intervals provided in parentheses. T

^*^ *p* < 0.05, ^**^ *p* < 0.01, ^***^ *p* < 0.001

**Table 3—Supplement: OLS Regressions on Satisfaction with Different Domains of Life**

**Using t0 as Reference: Pooled Model**

|  | Life | Family | Contacts |
| --- | --- | --- | --- |
| Age 65-79 | 0.32^***^ | 0.37^***^ | 0.27^***^ |
|  | [0.17,0.47] | [0.20,0.54] | [0.12,0.43] |
| t1 | -0.76^***^ | -0.54^***^ | -1.34^***^ |
|  | [-0.81,-0.71] | [-0.60,-0.48] | [-1.41,-1.26] |
| t2 | -0.71^***^ | -0.58^***^ | -1.51^***^ |
|  | [-0.76,-0.66] | [-0.64,-0.52] | [-1.58,-1.44] |
| t3 | -0.48^***^ | -0.36^***^ | -1.06^***^ |
|  | [-0.52,-0.43] | [-0.41,-0.30] | [-1.13,-1.00] |
| t1 * Age 65-79 | 0.32^***^ | 0.04 | 0.60^***^ |
|  | [0.21,0.43] | [-0.10,0.18] | [0.44,0.76] |
| t2 * Age 65-79 | 0.19^*^ | -0.09 | 0.50^***^ |
|  | [0.02,0.35] | [-0.28,0.09] | [0.31,0.69] |
| t3 * Age 65-79 | 0.25^**^ | 0.10 | 0.49^***^ |
|  | [0.10,0.40] | [-0.06,0.26] | [0.33,0.66] |
| N (persons) | 3039 | 3036 | 3039 |
| N (observations) | 11697 | 11530 | 11704 |
| R2 | 0.13 | 0.10 | 0.15 |

Note: Coefficients stem from OLS regressions with clustered, robust standard errors; confidence intervals provided in parentheses. The models additionally include gender, partnership status, employment status, health and size of place of residence.

^*^ *p* < 0.05, ^**^ *p* < 0.01, ^***^ *p* < 0.001

**Table 4—Supplement: Comparison of Coefficients Across Measurement Occasions and Life Domain by Age Groups**

| \|  \| **40 to 64 yrs.** \| \| **65 to 79 yrs.** \| \| \| --- \| --- \| --- \| --- \| --- \| \|  \| χ2 \| p-value \| χ2 \| p-value \| \| **t0 vs. t1** \|  \|  \|  \|  \| \| Life vs. family satisfaction \| 62.67 \| 0.00 \| 0.79 \| 0.37 \| \| Life vs. contact quality satisfaction \| 338.06 \| 0.00 \| 20.47 \| 0.00 \| \| Family vs. contact quality satisfaction \| 442.68 \| 0.00 \| 9.80 \| 0.00 \| \|  \|  \|  \|  \|  \| \| **t1 vs. t2** \|  \|  \|  \|  \| \| Life vs. family satisfaction \| 7.63 \| 0.01 \| 1.09 \| 0.30 \| \| Life vs. contact quality satisfaction \| 40.10 \| 0.00 \| 4.87 \| 0.03 \| \| Family vs. contact quality satisfaction \| 11.96 \| 0.00 \| 1.26 \| 0.26 \| \|  \|  \|  \|  \|  \| \| **t1 vs. t3** \|  \|  \|  \|  \| \| Life vs. family satisfaction \| 11.69 \| 0.00 \| 0.15 \| 0.70 \| \| Life vs. contact quality satisfaction \| 0.00 \| 0.96 \| 0.23 \| 0.63 \| \| Family vs. contact quality satisfaction \| 6.36 \| 0.01 \| 0.63 \| 0.43 \| |
| --- | --- | --- | --- | --- | --- | --- | --- | --- | --- | --- | --- | --- | --- | --- | --- | --- | --- | --- | --- | --- | --- | --- | --- | --- | --- | --- | --- | --- | --- | --- | --- | --- | --- | --- | --- | --- | --- | --- | --- | --- | --- | --- | --- | --- | --- | --- | --- | --- | --- | --- | --- | --- | --- | --- | --- | --- | --- | --- | --- | --- | --- | --- | --- | --- | --- | --- | --- | --- | --- | --- | --- | --- | --- | --- | --- | --- | --- | --- | --- | --- |

Note: Stata’s suest command was used to compare regression coefficients across life domains and time points (taken from Table 2).

**Table 5—Supplement**: FIML Estimation on Satisfaction with Different Areas of Life (Before and During the Corona Pandemic) by Age Group

|  | Life  40-64 | 65-79 | Family  40-64 | 65-79 | Contacts  40-64 | 65-79 |
| --- | --- | --- | --- | --- | --- | --- |
|  |  |  |  |  |  |  |
| t0 | 0.77^***^ | 0.42^***^ | 0.56^***^ | 0.62^***^ | 1.38^***^ | 0.74^***^ |
|  | [0.74,0.81] | [0.34,0.50] | [0.52,0.60] | [0.51,0.72] | [1.33,1.43] | [0.63,0.85] |
| t2 | 0.08^***^ | -0.10 | -0.01 | -0.09 | -0.11^***^ | -0.27^***^ |
|  | [0.03,0.12] | [-0.23,0.03] | [-0.06,0.04] | [-0.25,0.07] | [-0.16,-0.05] | [-0.41,-0.12] |
| t3 | 0.32^***^ | 0.21^***^ | 0.20^***^ | 0.35^***^ | 0.34^***^ | 0.14^*^ |
|  | [0.27,0.37] | [0.09,0.33] | [0.15,0.26] | [0.20,0.50] | [0.28,0.40] | [0.00,0.28] |
| Women | -0.06 | -0.04 | -0.05 | -0.14 | 0.15^***^ | 0.10 |
|  | [-0.12,0.00] | [-0.19,0.12] | [-0.11,0.02] | [-0.32,0.03] | [0.08,0.21] | [-0.07,0.27] |
| Partnered | 0.21^***^ | 0.14 | 0.50^***^ | 0.51^***^ | -0.25^***^ | -0.23^*^ |
|  | [0.14,0.27] | [-0.04,0.31] | [0.43,0.58] | [0.30,0.72] | [-0.32,-0.18] | [-0.43,-0.04] |
| University degree | -0.02 | -0.05 | -0.02 | -0.03 | -0.13^***^ | -0.08 |
|  | [-0.08,0.05] | [-0.22,0.13] | [-0.10,0.05] | [-0.23,0.17] | [-0.20,-0.06] | [-0.27,0.12] |
| Working | 0.16^*^ | -0.05 | 0.03 | -0.12 | 0.12 | 0.01 |
|  | [0.03,0.29] | [-0.21,0.12] | [-0.11,0.17] | [-0.32,0.08] | [-0.01,0.25] | [-0.17,0.18] |
| Health | -0.40^***^ | -0.29^***^ | -0.34^***^ | -0.25^***^ | -0.18^***^ | -0.17^**^ |
|  | [-0.44,-0.35] | [-0.39,-0.19] | [-0.39,-0.30] | [-0.36,-0.13] | [-0.22,-0.14] | [-0.28,-0.06] |
| Town size >50,000 | -0.10^**^ | -0.04 | -0.09^*^ | -0.01 | -0.07^*^ | 0.03 |
|  | [-0.16,-0.04] | [-0.20,0.11] | [-0.15,-0.02] | [-0.19,0.17] | [-0.14,-0.01] | [-0.15,0.20] |
| Constant | 5.74^***^ | 6.24^***^ | 5.61^***^ | 5.67^***^ | 4.94^***^ | 5.72^***^ |
|  | [5.53,5.95] | [5.80,6.69] | [5.39,5.83] | [5.16,6.19] | [4.73,5.15] | [5.21,6.23] |
| N (persons) | 5803 | 727 | 5803 | 727 | 5803 | 727 |
| N (observations) | 17639 | 2342 | 17639 | 2342 | 17639 | 2342 |
